# An extract from the frass of swallowtail butterfly (*Papilio machaon*) larvae inhibits HCT116 colon cancer cell proliferation but not other cancer cell types

**DOI:** 10.1186/s12864-023-09841-0

**Published:** 2023-12-04

**Authors:** Miho Nakano, Takuma Sakamoto, Yoshikazu Kitano, Hidemasa Bono, Richard J. Simpson, Hiroko Tabunoki

**Affiliations:** 1 Cooperative Major in Advanced Health Science, Graduate School of Bio-Applications and System Engineering, Tokyo University of Agriculture and Technology, Tokyo, Fuchu 183-8509 Japan; 2 Department of Science of Biological Production, Graduate School of Agriculture, Tokyo University of Agriculture and Technology, Tokyo, Japan; 3 Department of Applied Biological Science, Tokyo University of Agriculture and Technology, 3-5-8 Saiwai- cho, Fuchu-shi, Tokyo, 183-8509 Japan; 4 Laboratory of Bio-DX, Genome Editing Innovation Center, Hiroshima University, 3-10-23 Kagamiyama, Higashi-Hiroshima City, 739-0046 Japan; 5 Laboratory of Genome Informatics, Graduate School of Integrated Sciences for Life, Hiroshima University, 3- 10-23 Kagamiyama, Higashi-Hiroshima City, 739-0046 Japan; 6 Department of Biochemistry and Chemistry, La Trobe Institute for Molecular Science (LIMS), School of Agriculture, Biomedicine and Environment, La Trobe University, Melbourne, VIC 3086 Australia; 7 Institute of Global Innovation Research, Tokyo University of Agriculture and Technology, 3-5-8 Saiwai-cho, Fuchu, Tokyo 183-8509 Japan

**Keywords:** Insect metabolites, Cytochrome P450 (*CYP*), *CYP6B2*, Chalcone, Transcriptome analysis, *Papilio machaon*, *Angelica Keiskei*

## Abstract

**Background:**

The frass of several herbivorous insect species has been utilised as natural medicines in Asia; however, the metabolite makeup and pharmaceutical activities of insect frass have yet to be investigated. Oligophagous Papilionidae insects utilise specific kinds of plants, and it has been suggested that the biochemicals from the plants may be metabolised by cytochrome P450 (*CYP*) in Papilionidae insects. In this study, we extracted the components of the frass of *Papilio machaon* larvae reared on *Angelica keiskei*, *Oenanthe javanica* or *Foeniculum vulgare* and examined the biological activity of each component. Then, we explored the expression of *CYP* genes in the midgut of *P. machaon* larvae and predicted the characteristics of their metabolic system.

**Results:**

The components that were extracted using hexane, chloroform or methanol were biochemically different between larval frass and the host plants on which the larvae had fed. Furthermore, a fraction obtained from the chloroform extract from frass of *A. keiskei*-fed larvae specifically inhibited the cell proliferation of the human colon cancer cell line HCT116, whereas fractions obtained from the chloroform extracts of *O. javanica*- or *F. vulgare*-fed larval frass did not affect HCT116 cell viability. The metabolites from the chloroform extract from frass of *A. keiskei*-fed larvae prevented cell proliferation and induced apoptosis in HCT116 cells. Next, we explored the metabolic enzyme candidates in *A. keiskei*-fed larvae by RNA-seq analysis. We found that the *A. keiskei*-fed larval midgut might have different characteristics from the *O. javanica*- or *F. vulgare*-fed larval metabolic systems, and we found that the *CYP6B2* transcript was highly expressed in the *A. keiskei*-fed larval midgut.

**Conclusions:**

These findings indicate that *P. machaon* metabolites might be useful as pharmaceutical agents against human colon cancer subtypes. Importantly, our findings show that it might be possible to use insect metabolic enzymes for the chemical structural conversion of plant-derived compounds with complex structures.

**Supplementary Information:**

The online version contains supplementary material available at 10.1186/s12864-023-09841-0.

## Background

Over one million species of insects have been recorded, and half of them are categorised as herbivorous insects that utilise plants [1, 2]. Herbivorous insects are categorised into three types: (1) monophagous insects that utilise only one species of host plant, (2) oligophagous insects that utilise a narrow range of host plants, and (3) polyphagous insects that utilise a broad range of host plants [2]. Polyphagous insects have the advantage of being able to feed on a balanced diet at any time and in any place, while monophagous and oligophagous insects can specialise in utilising toxic metabolites from their host plants to protect themselves from predators. Heliconian butterflies, which are monophagous insects, produce cyanogenic glucoside, which is metabolised from their host plants in the Passifloraceae family [3]. Furthermore, troidine swallowtails, which belong to Papilionidae and feed on plants of the Aristolochiaceae family, sequester aristolochic acid from their host plants [4]. These monophagous and oligophagous insects are therefore difficult for predators to eat because they are unpalatable [5].

Frass from herbivorous insects has been used as a source of natural medicine in several countries. Insect frass contains plant-derived ingredients whose chemical structures were changed by insect metabolism. Thus, ingredients from insect frass may have enhanced biological activity compared with the biological activity of the metabolites that the insects originally consumed from plants. Some medicinal herbs and frass from a stick insect, *Eurycnema versifasciata*, are used to treat a variety of ailments, including diarrhoea, asthma, upset stomach and muscular pain, in Malaysia and China [6, 7]. In addition, ingredients included in the *Bombyx mori* larval frass are thought to stimulate the adrenal gland and to decrease blood cholesterol content [8]. These examples demonstrate that the frass of several phytophagous insect species has been used as pharmaceutical agents in several countries to treat human diseases. The therapeutic effects of insect frass may depend on the insects’ host plants, with the metabolites found in frass being converted into more effective metabolites than those found in the host plant tissues through the process of insect metabolism [9]. However, the process by which insect metabolism changes the biological activity of host plant metabolites have been obscured.

Cytochrome P450 (CYP), a drug-metabolising enzyme that is highly conserved in most organisms, has potential for biotechnological use because of its high diversity of catalysed reactions, and because high productivity is needed for such biotechnological applications [10, 11]. Most Papilionidae family members, including polyphagous and oligophagous insects, feed on furanocoumarin-containing plants [12]. Furthermore, oligophagous Papilionidae can metabolise furanocoumarins more efficiently than polyphagous insects by means of their CYP enzymes [13, 14]. Thus, CYP enzymes from oligophagous Papilionidae are a potential resource for biotechnology; however, their metabolic function in processing host plant metabolites, other than furanocoumarins, has yet to be investigated.

**Fig. 1:**
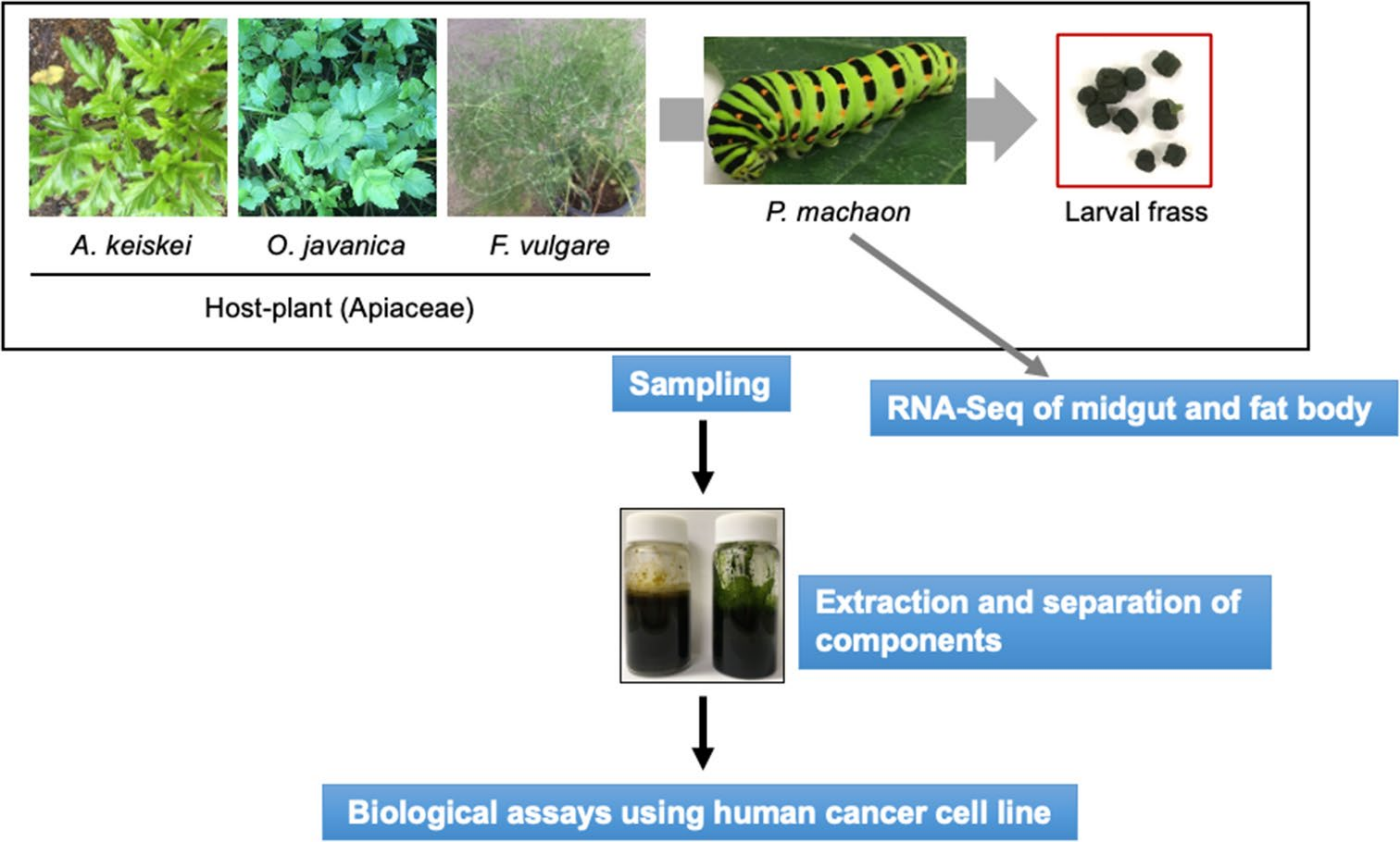
Outline of this study. *P. machaon* larvae ate three kinds of host plants, and then the metabolites were extracted from the frass of each host plant group using three kinds of organic solvents, and the fractions were separated using open column chromatography. The fractions were examined for biological activity using human cancer cell lines

Larvae of *Papilio machaon*, which belongs to the Papilionidae family, are specialists of Apiaceae family plants, such as *Angelica keiskei*, *Oenanthe javanica* and *Foeniculum vulgare* (commonly known as fennel). These plants have been used as traditional medicine for a wide range of ailments, with their extracts showing various biological activities, including antitumour, anti-inflammatory, antioxidant and antiviral effects [15–17]. Therefore, we speculated that the metabolites of *P. machaon* larval frass, which includes host plant-derived metabolites, may contain biological activities of interest [18]. In this study, we aimed to evaluate the biological activity of the metabolites from the frass of *P. machaon* larvae fed on *A. keiskei*, *O. javanica* or *F. vulgare* using a human cancer cell line (HCT116), and we analysed the metabolic process of these metabolites in *P. machaon* larvae (Fig. 1).

## Results

### *Fraction derived from the chloroform extract of frass of* P. machaon *larvae fed with* A. keiskei *specifically inhibits HCT116 cell viability*

**Fig. 2:**
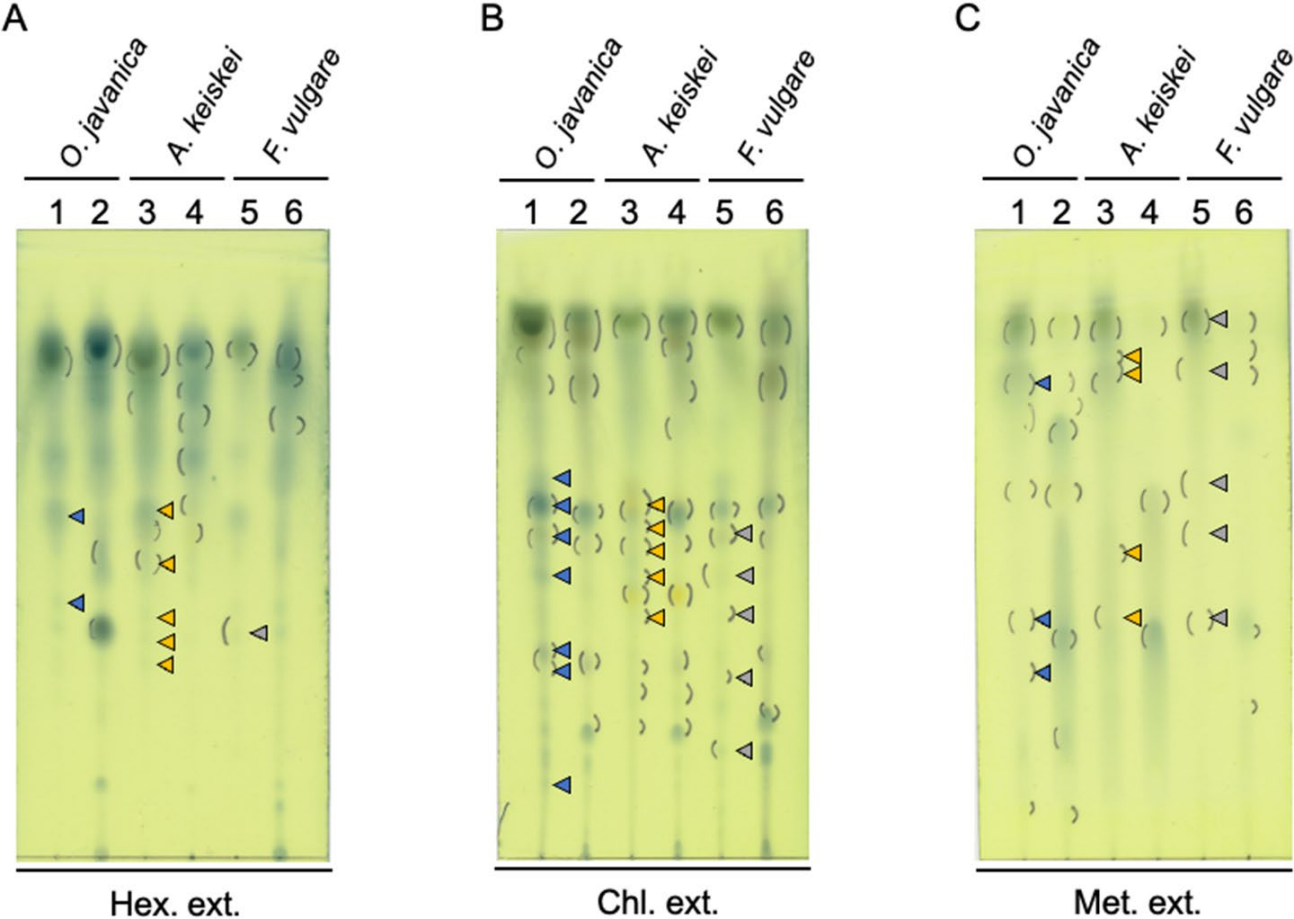
Comparison of the metabolites included in each extract using TLC analysis. The different spots between extracts from frass and extracts from host plants are shown as arrowheads for Hex. ext. **(A)**, Chl. ext. **(B)** and Met. ext. **(C)**

First, we produced extracts from the frass of *P. machaon* larvae fed on *A. keiskei*, *O. javanica*, or *F. vulgare* using n-hexene, chloroform, and methanol (Fig. 1). Then, we compared the metabolic products from both *P. machaon* frass and the leaves of *A. keiskei*, *O. javanica*, or *F. vulgare* using thin-layer chromatography (TLC) plate chromatograms (Fig. 2, Table S1). Staining the TLC plate chromatograms with sodium phosphomolybdate solution revealed two spots with calculated Relative to front (Rf) values ranging from 0.55 to 0.40 in the n-hexane extract (Hex. ext.) of frass from *O. javanica*-fed larvae (Fig. 2A lane 1, blue arrowheads), and we detected five spots with Rf values ranging from 0.55 to 0.31 in the Hex. ext. of frass from *A. keiskei*-fed larvae (Fig. 2A lane 3, orange arrowheads), and one spot with an Rf value of 0.36 in the Hex. ext. of frass from *F. vulgare*-fed larvae (Fig. 2A lane 5, grey arrowheads). Seven spots with Rf values from 0.61 to 0.13 were detected in the chloroform extract (Chl. ext.) of frass from *O. javanica*-fed larvae (Fig. 2B lane 1, blue arrowheads), and we detected five spots with Rf values ranging from 0.57 to 0.39 in the Chl. ext. of frass from *A. keiskei*-fed larvae (Fig. 2B lane 3, orange arrowheads), and five spots of Rf values from 0.59 to 0.18 in the Chl. ext. of frass from *F. vulgare*-fed larvae (Fig. 2B lane 5, grey arrowheads). Three spots with Rf values from 0.76 to 0.30 were detected in the methanol extract (Met. ext.) of frass from *O. javanica*-fed larvae (Fig. 2C lane 1, blue arrowheads), and we detected five spots with Rf values from 0.85 to 0.38 in the Met. ext. of frass from *A. keiskei*-fed larvae (Fig. 2C lane 3, orange arrowheads), and five spots of Rf values from 0.86 to 0.38 in the Met. ext. of frass from *F. vulgare*-fed larvae (Fig. 2C lane 5, grey arrowheads). We did not find these spots in the Hex., Chl. and Met. extracts of leaves from the *A. keiskei*, *O. javanica*, and *F. vulgare* plants (Fig. 2A and C, lane 2,4,6). In a previous study, the Chl. ext. of frass from *Poncirus trifoliata*-fed *Papilio xuthus* larvae inhibited cell viability of the human pancreatic cancer cell line MIA PaCa2 [19].

**Fig. 3:**
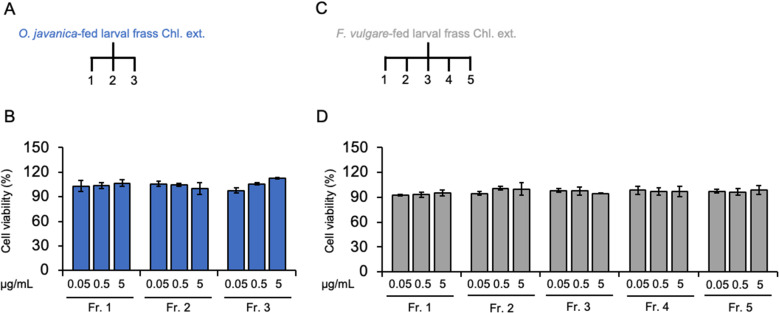
Cell viability of HCT116 cells treated with fractions. **(A, B)** Fractions separated from Chl. ext. of *O. javanica*-fed larval frass **(A)** and their effect on HCT116 viability **(B)**. **(C, D)** Fractions separated from Chl. ext. of *F. vulgare*-fed larval frass **(C)** and their effect on HCT116 viability **(D)**. Cell viability was measured using the WST-8 assay. Error bars represent the mean ± SD from three biological replicates

Thus, we chose the Chl. extracts produced from the frass of *P. machaon* larvae that had been fed on *A. keiskei*, *O. javanica* or *F. vulgare* for use in subsequent investigations. These extracts were separated using open column chromatography to obtain an active fraction for use in testing the biological activity of the extracts against the human cancer cell line HCT116. Next, we evaluated the cell viability under exposure to these fractions using the HCT116 cell line by WST-1 or WST-8 assay. The Chl. ext. of frass from *P. machaon* larvae that fed on *O. javanica* was separated into three fractions, and these fractions did not decrease viability in the HCT116 cells (Fig. 3A and B). Similarly, the Chl. ext. of frass from *P. machaon* larvae that fed on *F. vulgare*, which was separated into four fractions, did not decrease viability in the HCT116 cells (Fig. 3C and D).

**Fig. 4:**
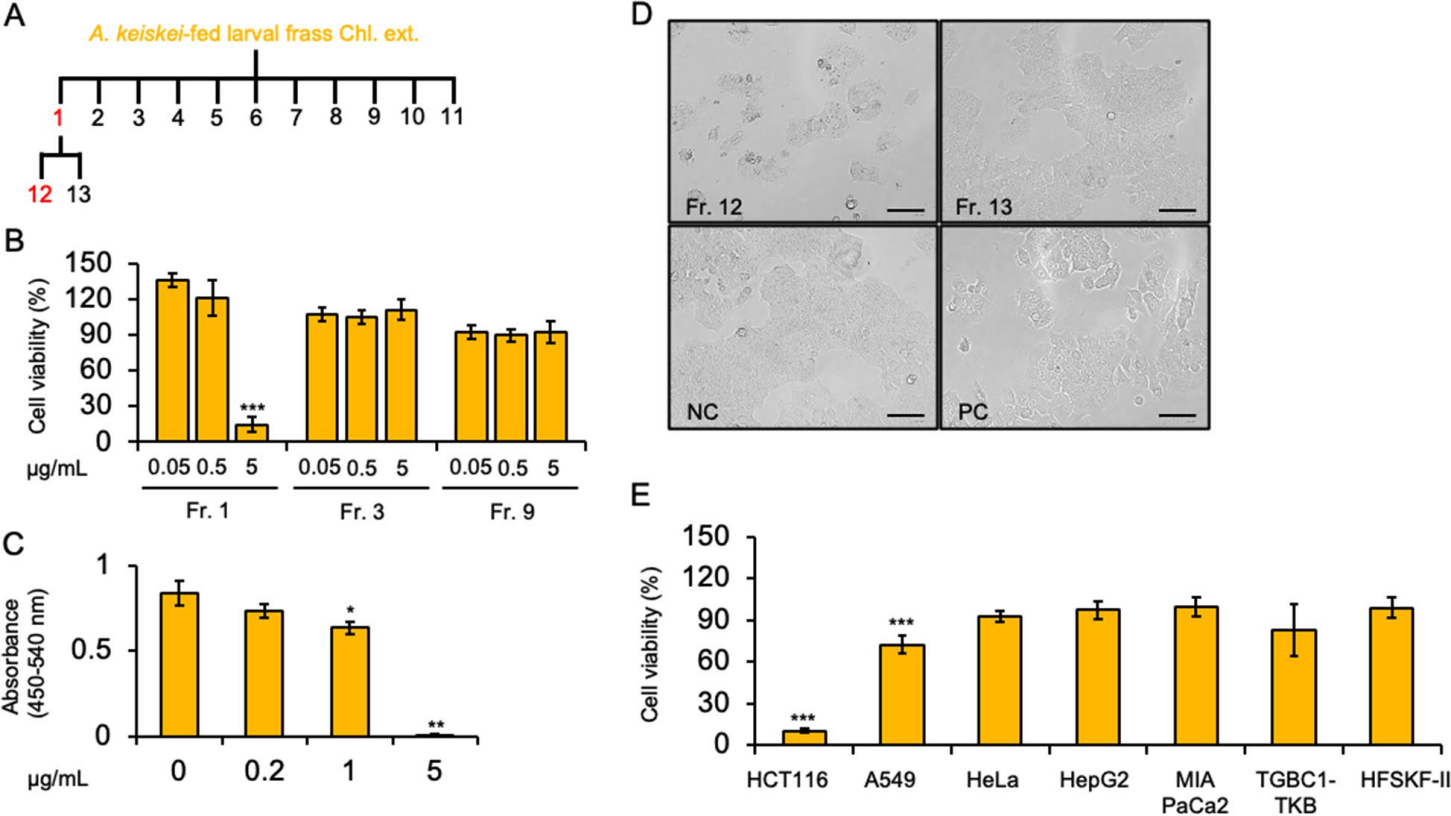
Biological activity on HCT116 by fractions separated from *A. keiskei*-fed larval frass. **(A)** Thirteen fractions separated from Chl. ext. using open column chromatography. **(B)** Cell viability of HCT116 cells treated with Fr. 1, Fr. 3 and Fr. 9. **(C)** BrdU assay in HCT116 cells after treatment with Fr. 1. The absorbance indicates inhibition of cell proliferation. **(D)** Morphological observation of HCT116 cells treated with 5 µg/mL of Fr. 12 or Fr. 13. DMSO (0.5%, v/v) was used as a negative control (NC), and 5 µM cisplatin (CDDP) was used as a positive control (PC). **(E)** Cell viability of A549 (lung cancer), HeLa (cervical cancer), HepG2 (hepatic cancer), MIA PaCa2 (pancreatic cancer), TGBC1TKB (gallbladder cancer) and HFSKF-II (human fibroblasts derived from foetal skin) cells treated with 5 µg/mL of Fr. 12. Cell viability was determined by WST-1 assay. Error bars represent the mean ± SD from three biological replicates. Significant differences from the control were analysed with Student’s t test, *P <0.05, **P <0.01, ***P <0.001. Scale bars = 100 μm

However, the Chl. ext. of frass from *P. machaon* larvae that fed on *A. keiskei* was separated into eleven fractions, and TLC analysis showed the different spots in the Fraction (Fr.) 1, 3, and 9 (Fig. S1A, orange arrowheads). Of these fractions, we found that Fr. 1 showed significantly decreased viability (10.4%) in HCT116 cells (Fig. 4A and B). Additionally, we measured proliferation of HCT116 cells by using the bromodeoxyuridine (BrdU) assay. We observed significantly decreased BrdU uptake in HCT116 cells treated with 5 µg/mL of Fr. 1 from the *A. keiskei*-fed larval frass (Fig. 4C).

Then, we separated Fr. 1 by open-column chromatography into two fractions (Fr.12 and 13). TLC analysis showed a different spot in Fr.12 compared with Fr.13 (Fig. S1B, red and black arrowheads). Next, we observed cell morphological changes in HCT116 cells treated with Fr. 12 or 13, and we found the cell density was decreased in HCT116 cells treated with Fr. 12 compared with Fr. 13, and the negative control (Fig. 4D). Subsequently, we evaluated the viability several human cancer cell lines using Fr. 12. The results indicated that Fr.12 significantly affected cell viability only in the HCT116 cell line, but not in the A549, HeLa, HepG2, MIA PaCa2, and TGBC1TKB cell lines. Interestingly, Fr. 12 did not affect the viability of HFSKF-II cells, a normal human fibroblast cell line (Fig. 4E). TLC analysis revealed the specific spot in Fr.12 compared with Fr.13 (Fig. S1B, red arrowhead). Therefore, Fr. 12 only strongly affected the colon cancer cell line.

### Examination of metabolites in larval food plants using the TUATinsecta database

Fr.12, which was extracted from the frass of *P. machaon* larvae that were fed *A. keiskei*, inhibited HCT116 cell viability, whereas the fractions from the frass of larvae that were fed *O. javanica* or *F. vulgare* had no effect on the HCT116 cell viability. Thus, we predicted that the chloroform extract frass from *A. keiskei*-fed larvae contained compounds that differ from compounds in the frass of larvae fed with *O. javanica* or *F. vulgare*. To examine the compounds of each food plant—*O. javanica*, *F. vulgare*, or *A. keiskei*—we utilised the TUAT-insecta database, which integrates information for insect-host plant, its ingredients and ingredient-biological function. We can get plant-metabolite information or related biological activity when we input the host plant’s name [20]. This interrogation revealed that the metabolites of *A. keiskei*, *O. javanica* and *F. vulgare* are mainly classified into 3 groups based on their chemical structures: phenolics, terpenes/steroids and alkaloids. Notably, chalcones, including phenolics and flavonoids, are found only in *A. keiskei* (Table 1).

**Table 1:** Grouping the metabolites from food plants using the TUATinsecta database

	*A. keiskei*	*O. javanica*	*F. vulgare*
Phenolics	22	4	24
Flavonoids	18	2	7
Chalcones	13	-	-
Non-flavonoids	4	2	17
Coumarins (Furanocoumarins)	4 (3)	-	2 (0)
Terpenes and steroids	1	2	14
Alkaloids	-	-	-

### *Mechanism of action of metabolites from the frass of* P. machaon *larvae fed with* A. keiskei *on HCT116 cells*

**Fig. 5:**
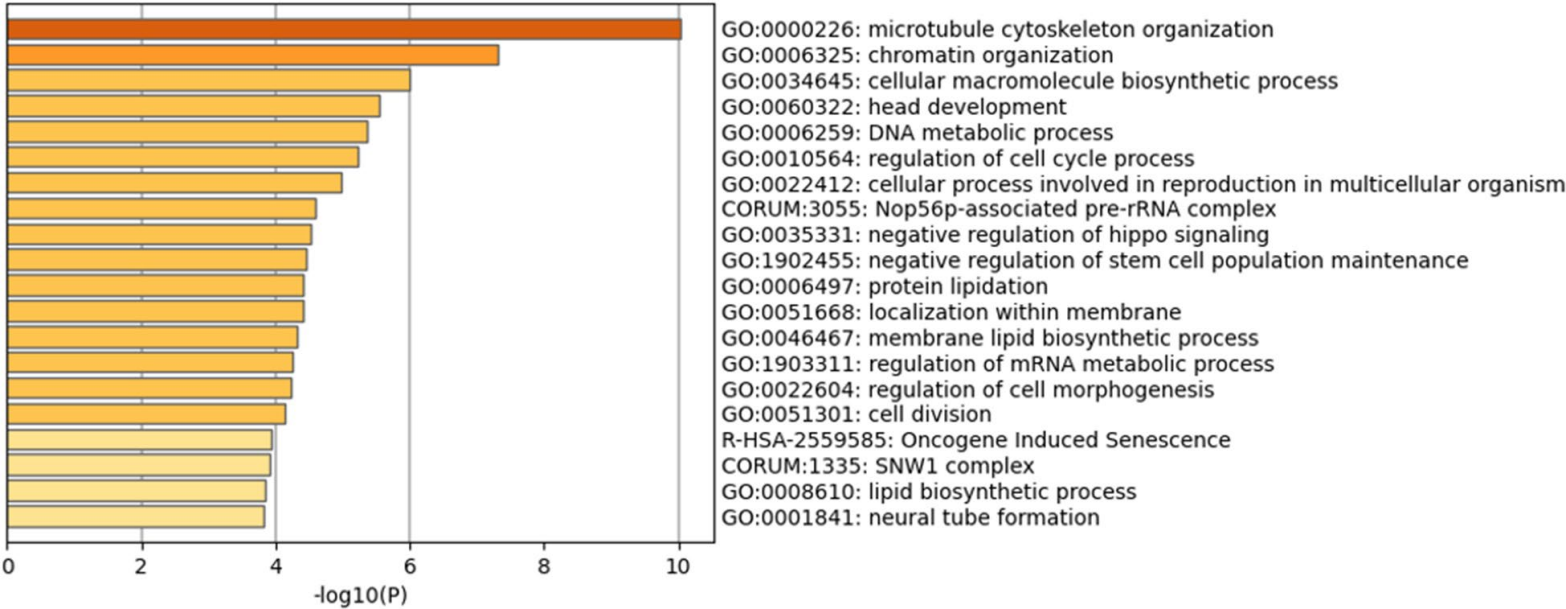
Gene enrichment analysis of DEGs in HCT116 cells treated with Fr. 12. Gene enrichment analysis of differentially expressed transcripts in HCT116 cells treated with Fr. 12 using Metascape. A bar graph for enriched terms across the input transcript lists; different coloured bars indicate different P values

To investigate the mechanism by which Fr. 12 decreases HCT116 cell viability, we employed transcriptome analysis using total RNA of the HCT116 cells after 48 h of treatment with 5 µg/mL of Fr. 12. Then, differentially expressed genes (DEGs) were identified based on the DEG analysis (false discovery rate, FDR < 0.05). All DEGs were shown in Table S2. Gene enrichment analysis showed that the DEGs were related to microtubule cytoskeleton organisation (GO: 0000226), a Gene Ontology (GO) term involved in cell division (Fig. 5). Additionally, we found that 19 genes out of the top 50 DEGs were those involved in HCT116 cell proliferation (15 genes), the cell cycle (6 genes) and apoptosis (9 genes) (Table 2 and Table S2). Therefore, our transcriptome analysis showed that Fr. 12 may induce inhibition of cell proliferation and apoptosis in HCT116 cells.

**Table 2:** The function of the top 50 DEGs in HCT116 cells treated with Fr. 12. DEGs

Rank	RefSeq_ID	Gene symbol	Gene function to human cancer cell line	Reference
6	XM_024451436	HSPBP1	Inhibition of cell proliferation in breast cancer by overexpression.	[21]
11	XM_006720559	NUSAP1	Inhibition of cell proliferation, and induction of apoptosis in colon cancer by knockdown.	[22]
12	XM_047440908	SETD4	Inhibition of cell proliferation and cell cycle at G1/S phase in breast cancer by knockdown.	[23]
14	XM_017009117	BTNL9	Inhibition of cell proliferation, colonization and cell cycle at G2/M phase, and induction of apoptosis in breast cancer.	[24]
18	NM_001369604	ST7	Overexpression in cell cycle arrest, and decreased expression in cell division.	[25]
19	XM_047434283	BANP	Inhibition of cell cycle and proliferation in breast cancer and colon cancer.	[26, 27]
22	XM_047447884	SASS6	Inhibition of cell cycle at G2/M phase and proliferation, induction of apoptosis in breast cancer and colon cancer by knockdown.	[28, 29]
24	NM_001350759	C1orf109	Inhibition of cell viability in breast cancer.	[30]
30	NM_080685	PTPN13	Inhibition of proliferation and colonization, and induction of apoptosis in renal cancer by increased expression.	[31]
34	XM_047443470	GPAT2	Inhibition of proliferation, and induction of apoptosis in breast cancer by knockdown.	[32]
38	NM_001077527	JRK	Inhibition of proliferation in breast cancer and colon cancer.	[33]
39	XM_047443471	GPAT2	Inhibition of proliferation, and induction of apoptosis in breast cancer by knockdown.	[32]
41	XM_047436491	TNRC6C	Inhibition of proliferation, and induction of apoptosis in papillary thyroid cancer by overexpression.	[34]
43	XM_047429511	ZNF84	Induction of proliferation by knockdown.	[35]
45	NM_021908	ST7	Inhibition of colonization and proliferation in prostate cancer and breast cancer.	[25]
46	NM_001346258	USP28	Induction of apoptosis in lung cancer by knockdown, and promotion of cell cycle and inhibition of apoptosis in pancreatic cancer by overexpression.	[36, 37]
47	NM_001369557	RPL17	Induction of apoptosis in colon cancer by knockdown.	[38]
48	NM_001281779	ZMYND8	Promotion of proliferation, migration and invasion in breast cancer and liver cancer.	[39, 40]
49	XM_005265382	SEMA3F	Inhibition of proliferation in oral cancer.	[41]

### Exploration of metabolic enzyme candidates that convert metabolites, including those from larval food plants, to active agents

**Fig. 6:**
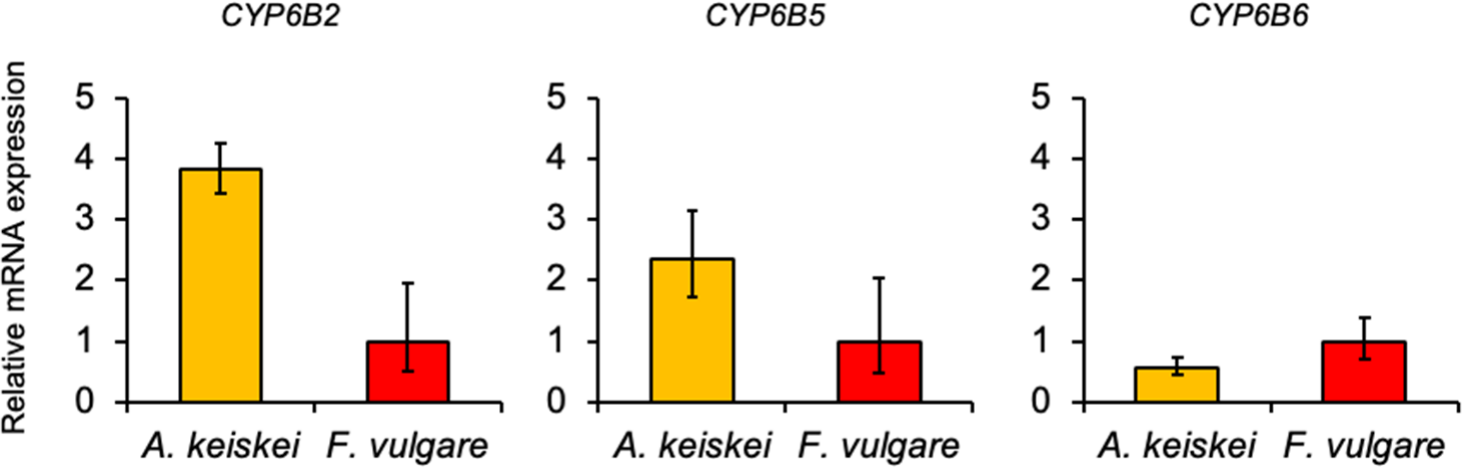
mRNA expression of *CYP* transcripts in the *P. machaon* larval midgut. The *CYP* transcripts expressed in the larval midgut fed on *A. keiskei* or *F. vulgare* were validated by RT-qPCR (*n* = 3). Relative Quantification (RQ) values represent the relative expression level compared to the larval midgut fed on *F. vulgare* as 1. rpL31 was used as endogenous control. The error bars represent the relative minimum / maximum expression levels relative to the mean RQ value from three biological replicates. **(A)*** CYP6B2*, **(B)*** CYP6B5*, **(C)*** CYP6B6*.

Extracts from the frass of *P. machaon* larvae fed with *A. keiskei* inhibited HCT116 cell viability. This indicates that metabolic enzymes in *P. machaon* convert metabolites, including those from its food plants, to active agents. Thus, we compared metabolic enzymes among the larvae that were fed *A. keiskei*, *O. javanica* or *F. vulgare* by using midgut and fat body transcriptome analysis. Importantly, CYP enzymes play a critical role in the metabolism of plant secondary metabolites [42]. Therefore, we focused on CYPs as metabolic enzymes that convert metabolites, including those of food plants, to active agents. First, we examined upregulated *CYP*s using their TPM value (Table S3). Next, we validated whether these *CYP* genes were upregulated or not by real-time quantitative PCR (RT-qPCR) as a biological replication, and we found that the expression of *CYP6B2* was upregulated in the midgut of *A. keiskei*-fed larvae compared with expression in the midgut of *F. vulgare*-fed larvae (Fig. 6A-C). Finally, we concluded that the *CYP6B2* transcript was upregulated in the midgut of *A. keiskei*-fed larvae.

## Discussion

In this study, we examined the biological activity of the metabolites in the frass extracts from *P. machaon* larvae fed *A. keiskei*, *O. javanica* or *F. vulgare* using a human cancer cell line (HCT116) and we analysed the metabolic processing of these metabolites in the larvae. *P. machaon* metabolites included in the chloroform extract from frass of *A. keiskei*-fed larvae inhibited cell viability and proliferation only in HCT116 cells. Notably, the fractions separated from the chloroform extract of *A. keiskei* leaves did not affect HCT116 viability (Fig. S2). Therefore, our results suggest that the metabolites from the frass might reflect a change in chemical structure and biological activity of *A. keiskei* metabolites when processed by the *P. machaon* larval metabolic system. *A. keiskei* contains several bioactive chalcones that are prenylated or geranylated at the 5’-position [15]. Xanthoangelol (5’-geranylated chalcone) induces apoptosis in the human leukaemia cell line K562 more strongly than isobavachalcone (5’-prenylated chalcone), whereas these compounds have no effect on human umbilical vein endothelial cells (HUV-EC) [43]. Xanthoangelol also suppresses the cell viability of the human leukaemia cells HL60, melanoma cells (CRL1579, AZ521) and human stomach cancer cells (KATO III); however, it does not inhibit A549 cell viability [44, 45]. Taken together, these findings indicate that the anticancer activity of 5’-prenylated chalcones changes depending on their modification. Therefore, we speculated that a *P. machaon* larval metabolite produced from an *A. keiskei* chalcone may have inhibited cell viability and proliferation, but only in the HCT116 cell line. The metabolism of chalcones in herbivorous insects awaits further investigation.

It has been reported that the 3’-prenylated chalcone xanthohumol is metabolised by CYP enzymes, and that its metabolites are excreted as faeces in rats [46, 47]. In this study, we found that the expression of *CYP6B2* transcript was upregulated in the midgut of *A. keiskei*-fed larvae compared with expression in the midgut of *F. vulgare*-fed larvae, and chalcones, including phenolics and flavonoids, were found only in *A. keiskei*. The CYP enzymes of the CYP6B subfamily from Papilionidae, including CYP6B1 and CYP6B3 from *P. polyxenes*, CYP6B17 and CYP6B21 from *P. glaucus*, and CYP6B33 from *P. multicaudata*, metabolise furanocoumarins [13, 14, 48, 49]. Of these, CYP6B1 enzyme metabolises more than 20 times more xanthotoxin (furanocoumarin) than that of CYP6B3 enzyme [49]. *CYP6B1* and *CYP6B3* mRNA are highly expressed in the larval midgut when xanthotoxin is administered to *P. polyxenes* larvae [50]. Although some Papilionidae that use furanocoumarin-containing plants have the *CYP6B2* gene, the metabolic products of CYP6B2 enzyme have not been identified [51]. Our results suggest that CYP6B2 enzyme in *P. machaon* larvae might relate to metabolise furanocoumarins and chalcones from the *A. keiskei* host plant. Therefore, chalcones from *A. keiskei* might be metabolised by CYP enzymes in *P. machaon* larvae as well as in mammals. However, we could not clarify how these *P. machaon* CYP enzymes metabolize plant-derived ingredients and decrease only HCT116 cell viability in this study.

In a future study, we will examine the function of these *P. machaon* CYP enzymes and determine whether *P. machaon* CYPs can be used as a bioprocess tool to change the chemical structure of plant ingredients.

## Conclusion

**Fig. 7:**
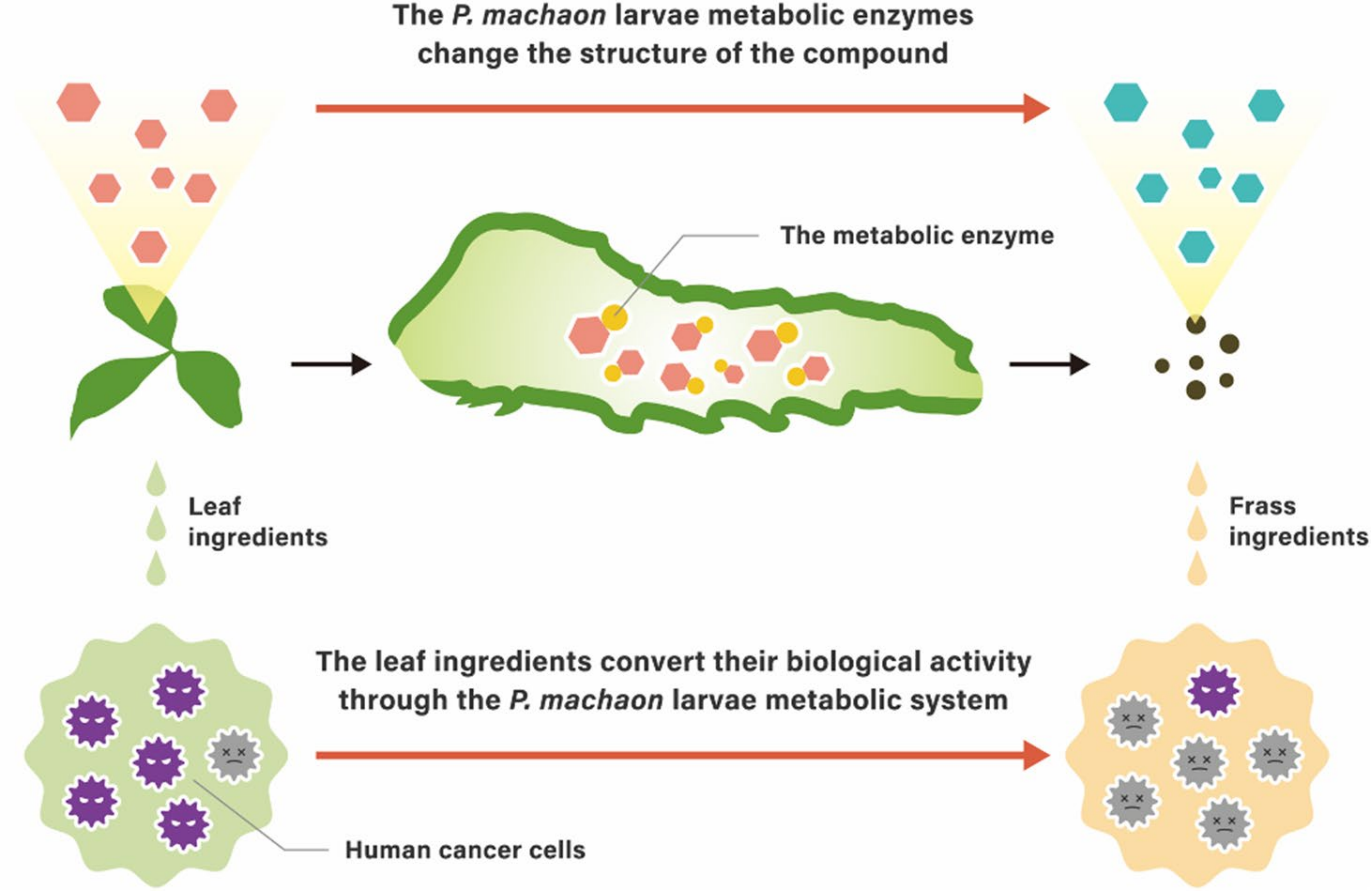
The predicted metabolic process for the metabolites derived from host plants. *P. machaon* larvae eat host plants, and their metabolic enzymes, CYPs, change the structure of compounds included in the host plant tissues. The metabolites from the host plants undergo conversion of their biological activity through the *P. machaon* larval metabolic process

The frass of *P. machaon* larvae reared on *A. keiskei* contain a metabolite that prevents cell division and induces apoptosis in human colon HCT116 cells. When we investigated the mechanism by which this metabolite was produced, the CYP6B2 enzyme appeared to be related to the metabolism of *A. keiskei* tissues containing chalcones. The metabolites from the plant undergo conversion of their biological activity through the *P. machaon* larval metabolic system (Fig. 7). These results indicate that the metabolites from *P. machaon* may be useful as pharmaceutical agents against human colon cancer. Additionally, it might be possible to use insect metabolic enzymes for the chemical structural conversion of plant-derived compounds with complex structures. Taken together, our results contribute to the future construction of a novel biotechnology system that entails creating potential pharmaceutical candidates using metabolic enzymes from *P. machaon* larvae.

## Methods

### Insects and sample collection

*P. machaon* larvae and eggs were collected at the Fuchu campus of the Tokyo University of Agriculture and Technology. *P. machaon* larvae were reared on the leaves of *A. keiskei* (open-pollinated variety), *O. javanica* (population), or *F. vulgare* (open-pollinated variety). These plants cultivated at the Fuchu campus of the Tokyo University of Agriculture and Technology. Larvae were maintained at 25 °C under a 16-h light/8-h dark cycle. The larval frass and host plant leaves were collected and stored at -30 °C until use.

### Extraction of metabolites from larval frass and leaves

We placed each sample on a glass petri dish and freeze-dried it with a lyophiliser (VD-250 F, TAITECH Co., Ltd., Saitama, Japan) for 24 h. We pulverised each sample and weighed it using a Mettler balance. We transferred the weighed sample to a 1000 mL Erlenmeyer flask and added 3 volumes of n-hexane (Hex; Wako Pure Chemical Industries, Ltd., Osaka, Japan). We allowed the mixture to sit for 24 h at room temperature (RT) and then filtered it. We transferred the pellet to a 1000 mL Erlenmeyer flask, added 3 volumes of Hex, left it for 24 h at RT, and then filtered it. We repeated this operation one more time and transferred the filtrate to an eggplant flask for evaporation. We transferred the pellet to a 1000 mL Erlenmeyer flask and added 3 volumes of chloroform (CHCl_3_; Wako Pure Chemical Industries, Ltd.) to the pellet. Then, we performed the same operation with Hex. We then transferred the pellet to a 1000 mL Erlenmeyer flask and added 3 volumes of methanol (MeOH; Wako Pure Chemical Industries, Ltd.) to the pellet. Then, we performed the same operation with Hex. We concentrated these filtrates, including Hex, CHCl_3_ or MeOH, by a rotary evaporator (N-1110 N, Tokyo Scientific Instruments Co., Ltd., Tokyo, Japan). Finally, we collected each extract in a screw tube (No. 7, Marem Co., Ltd., Osaka, Japan), kept the extracts in a vacuum desiccator for 3–5 days to completely remove each organic solvent and then weighed the obtained extract. We shielded the screw tubes containing the extracts from light with aluminium foil and stored them at 4 °C until use. We referred to the extract with n-hexane as Hex. ext., the extract with CHCl_3_ as Chl. ext., and the extract with MeOH as Met. ext. Table S4 shows the weight of samples before and after freeze-drying and the weight of their extracts.

### Thin-layer chromatography (TLC)

To separate and compare the metabolites from the larval frass or the host plants, each extract dissolved with extraction solvent at 10 mg/mL was applied to a silica gel TLC aluminium plate (Merck®, Darmstadt, Germany) in 1 µL (chloroform extract) or 2 µL (hexane and methanol extract) using a glass capillary. A mixed solvent was used as the eluent: chloroform-methanol (15:1, v/v) for the hexane extract, chloroform-methanol (10:1, v/v) for the chloroform extract and acetic acid-butanol-H_2_O (1:8:1, v/v) for the methanol extract. For detection of spots, we first used UV light to mark the spots (256 nm; left, 366 nm; right). Next, the plates were stained with sodium phosphomolybdate n-hydrate in ethanol (9%, v/v) to detect spots without UV exposure. We used the position of each spot as a relative to the front (Rf) value. The Rf value was calculated as follows: distance from baseline travelled by the solute divided by distance from baseline travelled by the solvent (solvent front).

### Separation of metabolites using open column chromatography

Chloroform extracts were separated on a silica gel (Kanto Chem., Tokyo, Japan) column (column size 30 × 300 mm and 170 mm height silica gel) with an eluting solvent of chloroform-methanol (10:1, v/v). These fractions dissolved in 300 mL solvent were collected in 26 mL glass test tubes to yield 11 fractions from the frass of *A. keiskei*-fed larvae, 3 fractions from the frass of *O. javanica*-fed larvae, 4 fractions from the frass of *F. vulgare*-fed larvae and 6 fractions from *A. keiskei* leaves based on the TLC profile. Fr. 1, separated from the chloroform extract from the frass of *A. keiskei*-fed larvae, inhibited HCT116 viability. Thus, Fr. 1 was further separated using a silica gel (FUJI SILYSIA Chemical LTD., Aichi, Japan) column (column size 15 × 300 mm, 170 mm height silica gel) and by eluting solvent of chloroform-methanol (18:1, v/v). The fractions dissolved in 60 mL of solvent were collected to yield 2 fractions based on the TLC profile.

### Cell culture

HCT116 (RCB2979), A549 (RCB0098), HeLa (RCB0007), HepG2 (RCB1886), MIA PaCa2 (RCB2094), TGBC1TKB (RCB1129) and HFSKF-II (RCB0698) cells were obtained from the Riken Cell Bank (Riken Tsukuba, Japan). A549, HCT116, HeLa, HepG2 and MIA PaCa2 cells were maintained in Dulbecco’s modified Eagle’s medium (DMEM, Nacalai Tesque, Kyoto, Japan) supplemented with 10% foetal bovine serum (FBS, Gibco, USA). TGBC1TKB cells were maintained in DMEM supplemented with 5% FBS. HFSKF-II was maintained in Ham’s F-12 (Nacalai Tesque, Kyoto, Japan) supplemented with 15% FBS. One hundred units/mL penicillin‒streptomycin (Fujifilm Wako Pure Chemical Corp., Osaka, Japan) was added to DMEM and Ham’s F-12. Cell lines were cultured at 37 °C in a humidified chamber containing 5% CO_2_.

### Cell viability assays

For the cell viability assays, cells were seeded in 96-well plates (HepG2: 700 cells per well; A549, HCT116, HeLa and TBC1TKB: 1000 cells per well; MIA PaCa2 and HFSKF-II: 2000 cells per well; experiment performed in triplicate). Then, after 24 h of incubation, medium containing each fraction dissolved in dimethyl sulfoxide (DMSO; Wako Pure Chemical Industries, Ltd.) was added to each well at final concentrations of 0.05, 0.5 and 5 µg/mL, whereas medium containing only DMSO was added to each control well at a final concentration of 0.5% (v/v). Forty-eight hours later, 10 µL/well WST-1 reagent (Takara Bio., Shiga, Japan) or WST-8 reagent (Doujin Chemical, Kumamoto, Japan) was added. After incubation for 4 h, the absorbance at 450 and 620 nm was measured using a microplate reader, iMark™ (Bio-Rad Laboratories Inc., Hercules, California, U.S.A.) or Gene5 (BioTek Instruments, Winooski, Vermont, U.S.A). The value of the absorbance at 620 nm, which was the control wavelength, was subtracted from the value of the absorbance at 450 nm. As a medium blank, 10 µL of WST-1 or WST-8 reagent was added to a well containing only medium without cells, and the average of the absorbance of this well was subtracted from all other values obtained. Finally, the cell viability was calculated using the following formula.

$$\begin{gathered} Cell{\text{ }}viability{\text{ }}\left( \% \right){\text{ }} = \hfill \\\,\,\,\,\,\,\,\,\,\,\,\,\,\,\,\,\,\,\,\,\,\,\,\,\,\,\,\,\,\,\,\,\,\,\,\,\,\,\,\,\,\,\,\,\left( {\left[ {Ab{s_{sample}} - Ab{s_{blank}}} \right]/\left[ {{\text{ }}Ab{s_{control}} - Ab{s_{blank}}} \right]} \right){\text{ }} \times {\text{ }}100 \hfill \\ \end{gathered}$$

### Cell proliferation assay

For the BrdU cell proliferation assay, 1000 HCT116 cells per well were cultured in a 96-well plate for 24 h. Then, the medium including Fr. 1 dissolved in DMSO was diluted with medium, and the medium was added to a well to a final concentration of 0.2, 1 and 5 µg/mL. After incubation for 24 h, 20 µL of BrdU reagent-containing medium was added to the measuring well, whereas 20 µL of BrdU reagent-free medium was added to a well as background. Finally, after culturing for 24 h, cell proliferation was evaluated using a BrdU assay kit (Abcam, Cambridge, U.K). according to the manufacturer’s protocols. The absorbance of background wells was subtracted from all other values obtained.

### Cell morphological observation

To assess the morphological changes in HCT116 cells after treatment with Fr. 12 and Fr. 13, the cells were cultured in a 24-well plate for 24 h. Then, Fr. 12, Fr. 13 (5 µg/mL), 0.5% (v/v) DMSO as a negative control or 5 µM cisplatin (CDDP; Fujifilm Wako Pure Chemical Corp., Osaka, Japan) as a positive control. Forty-eight hours later, the cell morphology was observed under a microscope, EVOS® FLoid® Cell Imaging Station (Thermo Fisher Scientific, Waltham, Massachusetts, U.S.A.)

### Comparisons of host plant components

To compare the chemicals contained in each host plant, we utilised TUAT-insecta [20], which is a database integrating information on herbivorous insects and their host plants with information on biological activity and chemicals. Information on *A. keiskei*, *O. javanica* and *F. vulgare* was obtained using their scientific names to search the TUAT-insecta database.

### RNA sequencing for HCT116 cells treated with Fr. 12

We purified total RNA from HCT116 cells treated with Fr. 12 (5 µg/mL, *n* = 3) or 0.1% (v/v) DMSO (*n* = 3) using TRIzol reagent (Thermo Fisher Scientific, USA) and the PureLink® RNA Extraction Kit (Thermo Fisher Scientific, Japan) according to the manufacturer’s protocol. Then, we used an Agilent TapeStation 2200 (Agilent Technologies, Santa Clara, CA) to assess the RNA quality. cDNA library construction of total RNA from these samples (100 ng) was carried out using the TruSeq® Stranded mRNA Library Preparation Kit (Illumina, Inc., San Diego, CA) according to the manufacturer’s instructions. Finally, the libraries (100 bp, paired-end) were sequenced using the Illumina NovaSeq6000 platform.

### Gene enrichment analysis in HCT116 cells treated with Fr. 12

To analyse the differentially expressed genes (DEGs) in HCT116 cells treated with Fr. 12, FASTQ files were assessed by TrimGalore! version 0.6.6 (https://www.bioinformatics.babraham.ac.uk/projects/trim_galore/). Then, these transcript data were mapped to the human transcript reference (GRCh38.p14) obtained from NCBI (accessed on 5th September 2022) using Salmon version 1.9.0 (http://salmon-tddft.jp/download.html) to obtain transcripts per kilobase million (TPM). Read count data were obtained from TPM by Salmon using the tximport package version 1.24.0 (https://github.com/thelovelab/tximport) [52]. All statistical analyses were performed using R version 4.2.1 (https://www.r-project.org/) with TMM normalization and voom package version 3.16. We extracted DEGs from HCT116 cells treated with Fr. 12 using a False Discovery Rate (FDR) < 0.05 [53]. Finally, we performed a functional analysis using the gene list of DEGs with the Metascape online tool (https://metascape.org/) with a default parameters [54]. The most statistically significant term within a cluster were chosen by Metascape to represent the cluster. Three samples in each group were used for RNA sequencing analysis.

### *RNA-Seq analysis in* P. machaon *larval midgut and fat body*

Total RNA from the midgut and fat body of *P. machaon* 5th larvae reared on *A. keiskei* (*n* = 1), *O. javanica* (*n* = 1) or *F. vulgare* (*n* = 1) were purified, and RNA-seq analysis was performed as mentioned above. All expressed genes were calculated using *P. machaon* reference transcripts downloaded from NCBI (ilPapMach1.1, accessed on 5th September 2022). To explore the *CYP* genes, we used the reference transcript data as follows: (1) translated the reference transcript into amino acid sequences using TransDecoder (https://github.com/TransDecoder/TransDecoder/releases) version 5.5.0; (2) the translated transcripts that having *CYP* domain (ID: PF00067, Protein Family (Pfam) database) extracted by HMMER program (http://hmmer.org) for obtaining *CYP* candidate IDs; (3) *CYP* multi-fasta file was created from the translated transcripts by extracting *CYP* candidate IDs list using makeblastdb with -parse_seqIds option in blastdbcmd functions of BLAST package (Basic Local Alignment Search Tool, version 2.13.0); (4) performed the functional gene annotation for identified *CYP* transcripts by BLAST search by blastp program with a cut-off *E*-value of 1e-10 using UniProt as a reference database; (4) Finally, we integrated the TPM from the Salmon results and the annotated these *CYP*s using TIBCO Spotfire v12.2.0 (TIBCO Spotfire, Inc., Palo Alto, CA; http://spotfire.tibco.com/better-world-donation-program/).

### *Evaluating mRNA expression of* CYP6B2, CYP6B5 *and* CYP6B6 *in* P. machaon *larval midgut by RT-qPCR*

Total RNA from the midgut of *P. machaon* 5th larvae reared on *A. keiskei* (*n* = 3), or *F. vulgare* (*n* = 3) were purified, then one microgram of total RNA was treated with DNase I (Invitrogen, Van Allen Way, Carlsbad, CA, USA), and then 500 ng of DNase-treated total RNA was used as a template for cDNA synthesis using a PrimeScript™ 1st strand cDNA Synthesis Kit (Takara co. Ltd., Tokyo, Japan) in accordance with the manufacturer’s instructions. Real-time quantitative PCR (RT‒qPCR) was performed in 20 µL reaction volumes with 0.13 µL of cDNA template and 0.4 µM each specific primers (Table 3) along with a KAPA SYBR Fast qRT‒PCR Kit (Nippon Genetics Co., Ltd., Tokyo, Japan) in accordance with the manufacturer’s instructions. RT‒qPCR was performed on a Step One Plus Real-Time PCR System (Applied Biosystems Foster City, CA) by following the Delta–Delta Ct method. *rpL31* was utilized as an endogenous reference against which RNA expression levels were standardized, and all data were calibrated against universal reference data. The relative expression level in comparison to a reference sample is represented by relative quantification (RQ) values. All sample sets were assayed in triplicate as biological replicates.

**Table 3:** Primers for RT-qPCR were used in this study

Gene name	Forward (5’-3’)	Reverse (5’-3’)
CYP6B2	TGGAATCCGCATTAAGGAAG	AAGGCGTGGTCATCCTGTAG
CYP6B5	CAACGCTCAAACGGACTACA	CGCTCAGGGTCAAACTTCTC
CYP6B6	TGCAGAACCACGCTATCAAG	AATCCATGTCGAGACCGAAC
rpL31	GTCGCGTAGACGCAATGATG	TTGATTGAGGCGACTGGCAC

### Data Availability

The RNA sequencing datasets generated and/or analysed during the current study are available in the Sequence Read Archive, DNA Data Bank of Japan repository, under the following accession IDs: HCT116 treated with Fr. 12 (SRA accession numbers: DRR428503, DRR428504, and DRR428505), HCT116 treated with DMSO (SRA accession numbers: DRR428506, DRR428507, and DRR428508), *P. machaon* larval midgut and fat body from larvae eating *A. keiskei*, (DRR428498, and DRR428497), *P. machaon* larval midgut and fat body from larvae eating *O. javanica* (DRR428502, and DRR428501), and *P. machaon* larval midgut and fat body from larvae eating *F. vulgare* (DRR428500, and DRR428499).

### Electronic supplementary material

Below is the link to the electronic supplementary material.

Supplementary Material 1

Supplementary Material 2

Supplementary Material 3

Supplementary Material 4

Supplementary Material 5

## Data Availability

The RNA sequencing datasets generated and/or analysed during the current study are available in the Sequence Read Archive, DNA Data Bank of Japan repository, under the following accession IDs: HCT116 treated with Fr. 12 (SRA accession numbers: DRR428503, DRR428504, and DRR428505), HCT116 treated with DMSO (SRA accession numbers: DRR428506, DRR428507, and DRR428508), *P. machaon* larval midgut and fat body from larvae eating *A. keiskei*, (DRR428498, and DRR428497), *P. machaon* larval midgut and fat body from larvae eating *O. javanica* (DRR428502, and DRR428501), and *P. machaon* larval midgut and fat body from larvae eating *F. vulgare* (DRR428500, and DRR428499).

## List of abbreviations

CYP: Cytochrome P450
TLC: Thin-layer chromatography
DEG: Differentially Expressed Gene
GO: Gene Ontology
KEGG: Kyoto Encyclopedia of Genes and Genomes
PCA: Principal component analysis
TPM: Transcripts per million
FDR: False Discovery Rate
BrdU: Bromodeoxyuridine
Rf: Relative to the front
RT-qPCR: Real-time quantitative PCR
rpL31: 60 S ribosomal protein L31
RQ: Relative quantification
