# Supplementary material for: An extract from the frass of swallowtail butterfly (Papilio machaon) larvae inhibits HCT116 colon cancer cell proliferation but not other cancer cell types
Source: BMC Genomics. 2023 Dec 4;24:735. doi: 10.1186/s12864-023-09841-0 (PMC10696813; doi:10.1186/s12864-023-09841-0)
Supplement: Supplementary file 1 — Supplementary Material 1 [file 12864_2023_9841_MOESM1_ESM.pdf]

An extract from the frass of swallowtail butterfly (*Papilio machaon*) larvae inhibits HCT116 colon cancer cell proliferation but not other cancer cell types

Miho Nakano[1], Takuma Sakamoto[2], Yoshikazu Kitano[3], Hidemasa Bono[4,5], Richard J. Simpson[6,7], and Hiroko Tabunoki[1,2,7]

[1]Cooperative Major in Advanced Health Science, Graduate School of Bio-Applications and System Engineering, Tokyo University of Agriculture and Technology, Tokyo, Fuchu, 183-8509, Japan
[2]Department of Science of Biological Production, Graduate School of Agriculture, Tokyo University of Agriculture and Technology, Tokyo, Japan
[3]Department of Applied Biological Science, Tokyo University of Agriculture and Technology, 3-5-8 Saiwai-cho, Fuchu-shi, Tokyo, 183-8509, Japan
[4]Laboratory of Bio-DX, Genome Editing Innovation Center, Hiroshima University, 3-10-23 Kagamiyama, Higashi-Hiroshima City 739-0046, Japan
[5]Laboratory of Genome Informatics, Graduate School of Integrated Sciences for Life, Hiroshima University, 3-10-23 Kagamiyama, Higashi-Hiroshima City 739-0046, Japan
[6]Department of Biochemistry and Chemistry, La Trobe Institute for Molecular Science (LIMS), School of Agriculture, Biomedicine and Environment, La Trobe University, Melbourne, VIC 3086, Australia
[7]Institute of Global Innovation Research, Tokyo University of Agriculture and Technology, 3-5-8 Saiwai-cho, Fuchu, Tokyo, 183-8509, Japan

*Corresponding author: Hiroko Tabunoki
Tel: +81-42-367-5613; Fax: +81-42-367-5613
E-mail: h_tabuno@cc.tuat.ac.jp

Supplementary figures

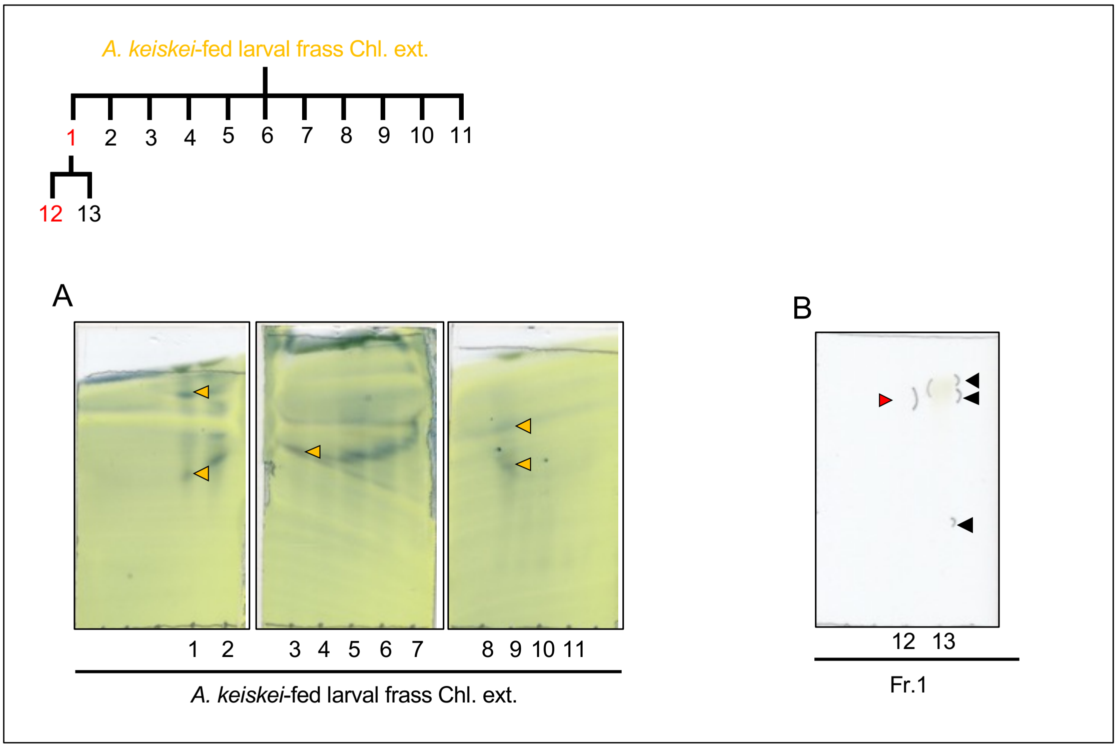

Figure S1 Comparison of fractions separated from chloroform extract of each larval frass group using TLC analysis. (A) Fractions from *A. keiskei*-fed larval frass Chloroform extract. (B) Fractions from *A. keiskei*-fed larval frass Chloroform extract, Fr.1. Arrowheads show different spots among the fractions.

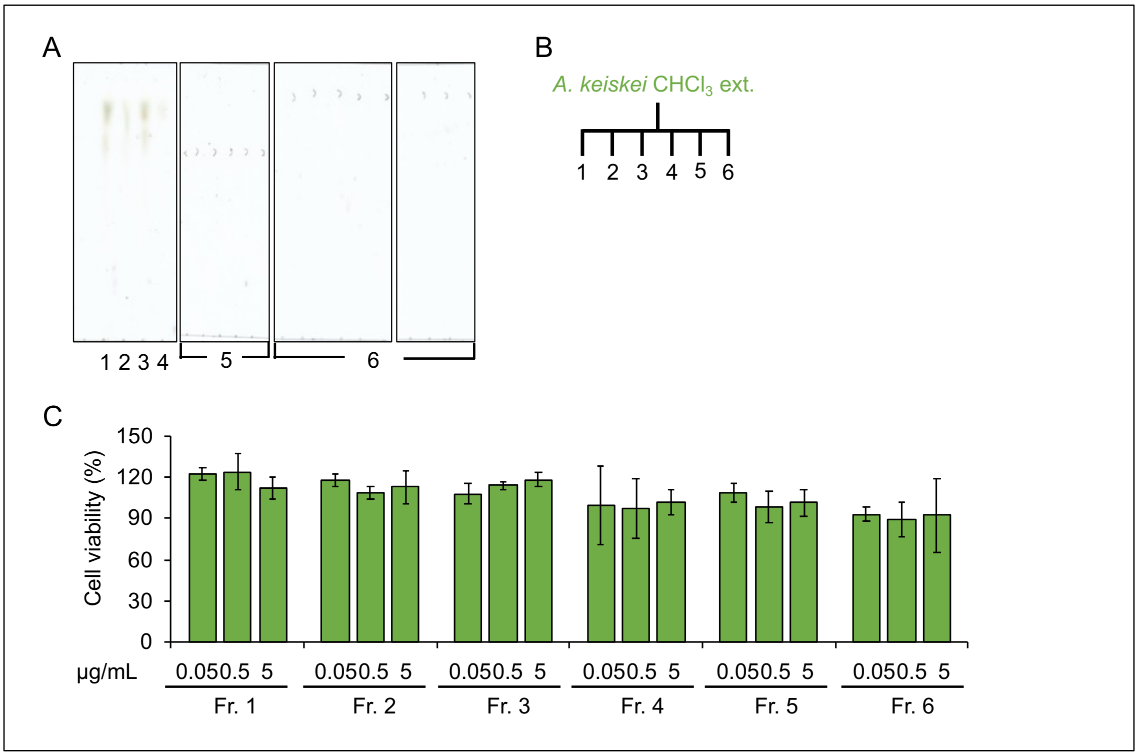

Figure S2 Cell viability of the fractions from chloroform extract of *A. keiskei* leaves. (A, B) Fractions separated from chloroform extract of *A. keiskei* leaves using open column chromatography were compared by TLC analysis (A) to obtain six fractions (B). (C) HCT116 viability when cells were treated with Fr. 1 to Fr. 6 from the *A. keiskei* leaf chloroform extract was determined by WST-1 assay. Error bars represent the mean ± SD from three biological replicates.
